# Case Report: Detection of Transferrin in a Dog Suspected of Having Cerebrospinal Fluid Rhinorrhea

**DOI:** 10.3389/fvets.2022.845809

**Published:** 2022-03-04

**Authors:** Kosuke Kinoshita, Hidetaka Nishida, Ryoji Kanegi, Yuya Nakamoto, Toshiyuki Tanaka, Shunsuke Shimamura, Kazuhito Kusumoto, Hideo Akiyoshi

**Affiliations:** ^1^Veterinary Medical Center, Osaka Prefecture University, Izumisano, Japan; ^2^Laboratory of Veterinary Surgery, Graduate School of Life and Environmental Sciences, Osaka Prefecture University, Izumisano, Japan; ^3^Neuro Vets Animal Neurology Clinic, Kyoto, Japan; ^4^Wakayama Inter Animal Hospital, Wakayama, Japan

**Keywords:** CSF leakage, dog, meningitis, transferrin, rhinorrhea

## Abstract

A 12-year-old Yorkshire terrier was referred for epileptic seizures and nasal discharge. The fluid was clear and serous. Cerebrospinal fluid (CSF) rhinorrhea was suspected, based on clinical signs and MRI findings. In humans, analysis of nasal secretions to determine the concentration of glucose and brain-type transferrin has been widely used clinically in order to confirm the presence of CSF rhinorrhea. The glucose concentration in the nasal discharge was 74 mg/dL. Serum-type and brain-type isoforms of transferrin were detectable in the nasal sample. The concentration of glucose and brain-type transferrin could be useful for diagnosing CSF rhinorrhea.

## Introduction

Cerebrospinal fluid (CSF) rhinorrhea is a type of CSF leakage (1). CSF leakage requires presence of a communication between the subarachnoid and extracranial space through the skull base (2). Causes are classified as traumatic and non-traumatic, and traumatic causes are more common in humans (3–5). Non-traumatic causes in humans are associated with neoplasia, inflammation, or congenital skull malformation, or are classified as idiopathic. In humans, diagnostic imaging including computed tomography (CT) and magnetic resonance imaging (MRI) have been used, and diagnosis can be made through nasal inspection and laboratory tests of the fluid (6). Analysis of nasal secretions in order to determine the concentration of glucose and brain-type transferrin has been widely used clinically in order to confirm the presence of CSF rhinorrhea.

Suspected or confirmed CSF rhinorrhea has been reported only sporadically in dogs (7, 8). In contrast to human medicine, diagnostic criteria for CSF rhinorrhea have not yet been established for dogs. We describe a case of suspected CSF rhinorrhea in a dog of which discharge was shown to contain brain-type transferrin.

## Case Presentation

A 12-year-old 1.5 kg entire male Yorkshire terrier was referred with a history of acute onset of seizures 1 day before admission. Generalized seizures occurred seven or eight times a day. Loss of consciousness was noted during seizure, and subsided in less than 1 min. After generalized seizures, post-ictal signs including transient circling and mild ataxia was observed. Nasal discharge was noted. The fluid was clear and serous. Hematology and serum biochemistry results were unremarkable. Mental status was alert during the neurological examination. Mydriasis of the right eye due to adhesion of the iris and the lens was observed. No other abnormalities were found upon neurological examination. Based on these findings, the differential diagnoses were neoplasia, inflammation, idiopathic, anomaly, or vascular disease. The lesion was localized to the cerebrum or diencephalon.

**Figure 1:**
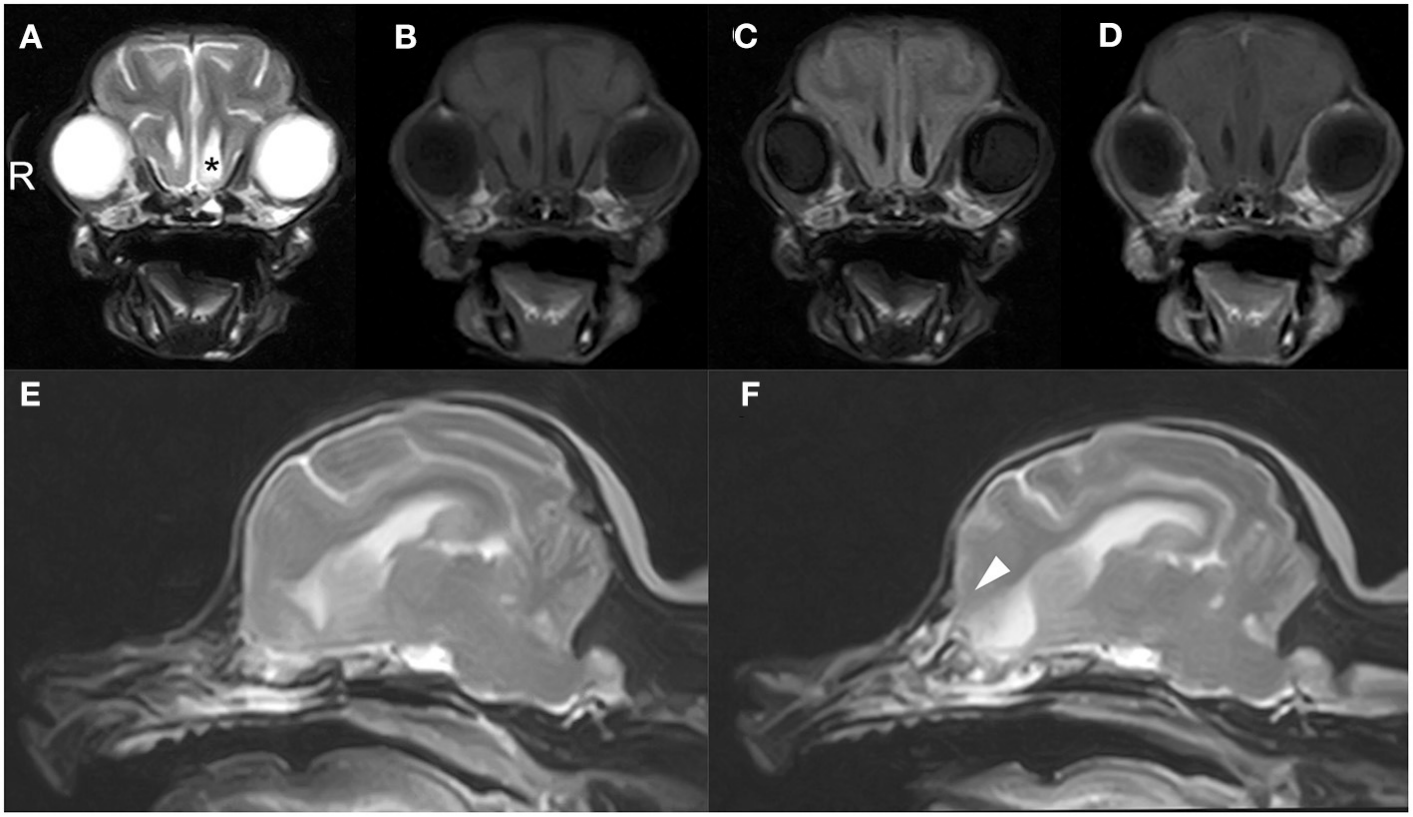
Brain magnetic resonance images of a 12-year-old Yorkshire terrier with epileptic seizures and nasal discharge **(A–F)**. Transverse T2 weighted **(A)**, T1 weighted **(B)**, FLAIR **(C)**, and T1 weighted post-contrast **(D)** images at the level of olfactory recess, and sagittal T2W1 image at the level of the right and left cribriform plate (**E, F**, respectively). Olfactory recess of the left lateral ventricular enlargement was observed (black asterisk) relative to the right lateral ventricle **(A)**. A defect of the left cribriform plate was observed (white arrowheads, **F**) relative to right cribriform plate **(E)**.

**Figure 2:**
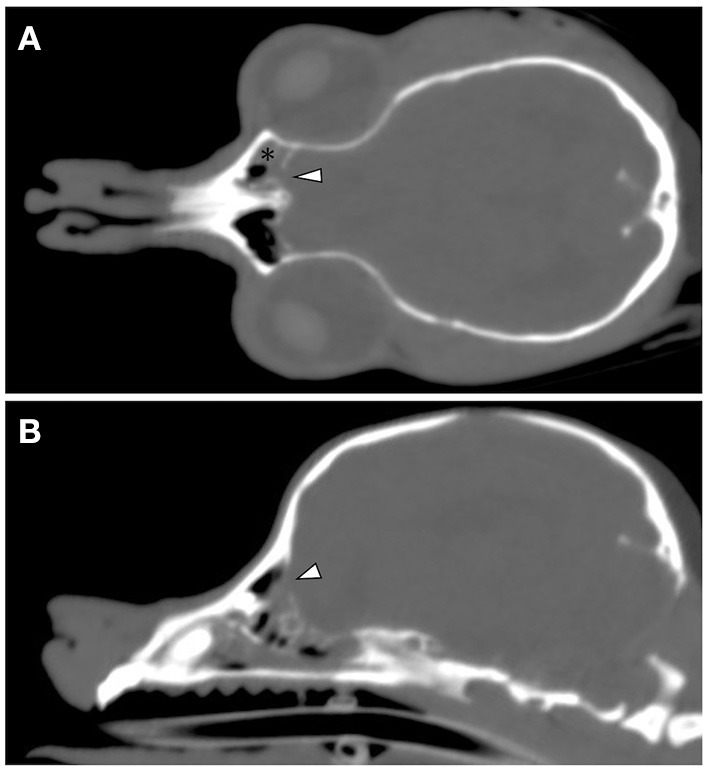
Cranial CT images of a 12-year-old Yorkshire terrier with epileptic seizures and nasal discharge **(A,B)**. On the coronal and sagittal bone condition images at the cribriform plate level (**A,B**, respectively), a defect of the left cribriform plate is noticeable (white arrowheads). On the coronal soft tissue images at the level of the optic nerve **(A)**, an irregular lesion in the left nasal cavity around the left cribriform plate is observed (black asterisk).

CT and MRI were undertaken to examine the cerebrum and diencephalon. Anesthesia was induced by propofol (MSD Animal Health, Tokyo, Japan) at a dose of 5.0 mg/kg and was maintained by isoflurane (MSD Animal Health). MRI was performed using a 0.4 T unit (APERTO Inspire version V5.0M; Hitachi Healthcare Systems, Osaka, Japan). The sequences included T2-weighted images (T2WI; TR, 2,800 ms; TE, 120 ms) of transverse and sagittal views, fluid-attenuated inversion recovery (FLAIR; TR, 6,900 ms; TE, 120 ms) of transverse view, T1-weighted images (T1WI; TR, 300 ms; TE, 14.3 ms) of transverse view, and postcontrast T1WI (gadoteridol, 0.2 mL/kg intravenous administration) of transverse view. Brain MRI revealed irregularities in the left cribriform plate compared to the right cribriform plate (Figures 1E,F). Also observed were the olfactory recess of the left lateral ventricular enlargement and irregular hyperintensity around the olfactory recess of the left lateral ventricle (Figures 1A–C). No signs of contrast enhancement were observed on postcontrast T1WI (Figure 1D). No other cerebral parenchymal lesion was observed. The CT scan (Activion16, Toshiba Medical Systems, Tochigi, Japan) was performed with a pitch of 0.9 mm, scan thickness of 0.5–2.00 mm, 100 mA, and 120 V. For contrast-enhanced imaging, the dog was administered with 2 mL/kg of non-ionic contrast medium (Ominipaque, GE Healthcare, Chicago, IL). Cranial CT revealed a defect of the left cribriform plate was observed (Figure 2A). No contrast enhancement was noticed. An irregular lesion in the left nasal cavity around the left cribriform plate was also observed (Figure 2B), for which the differential diagnosis was neoplasia, inflammation, or meningoencephalocele. No CSF tap was performed, because of malformation of the occipital bone. After CT and MRI examination, a sample of persistent nasal discharge was collected for cytological examination and for bacterial culturing. The bacterial culture results were negative, and cytologic evaluation showed non-infectious inflammation. Treatment was started with phenobarbital (4 mg/kg, BID).

No epileptic fits were observed in the 2 months after phenobarbital was started. No neurological abnormalities were detected. The serous nasal discharge persisted. On the 68th day, CT and MRI were repeated to examine changes in the olfactory recess. No remarkable changes were noted in a CT/MRI scan in the lesion, relative to the previous scan. Cytologic examination of the nasal discharge found many degenerated neutrophils and phagocyted cocci, indicating pyogenic infectious inflammation. *Streptococcus* sp. was detected in the bacterial culture test. Based on the examination of nasal discharge and on imaging suggesting destruction of the cribriform plate, CSF leakage and meningoencephalitis were suspected.

**Figure 3:**
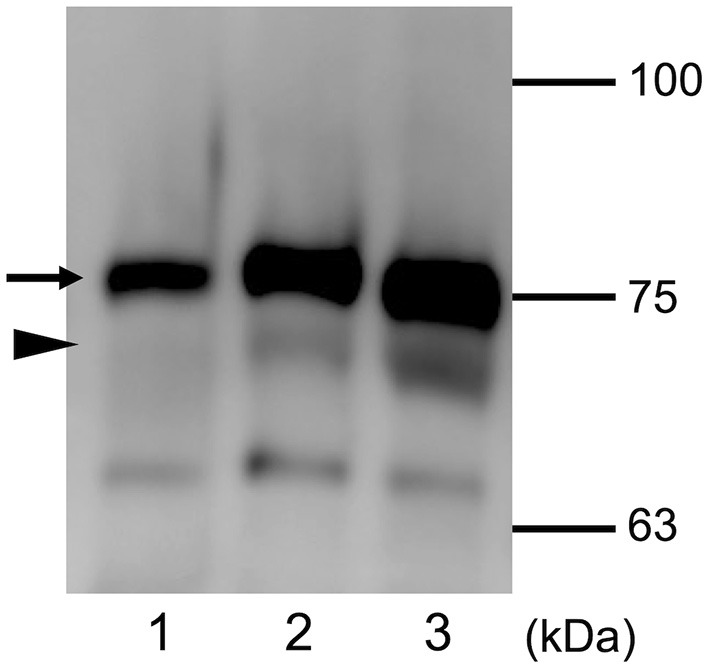
Expression of transferrin detected using Western blot analysis. Distinct protein patterns are seen with serum (*lane 1*) and CSF (*lane 2*) of a control dog, and nasal leaked fluid from the present patient (*lane 3*). The black arrow indicates the approximate position of serum transferrin, and black arrowhead indicates the approximate position of brain-type transferrin. The molecular masses of the size markers are shown on the right.

The glucose concentration in the nasal discharge of the dog was 74 mg/dL and CSF leakage was suspected, and further analysis was performed to check. Serum and CSF from a healthy dog (Beagle, 1 year old, female) were used as controls. This procedure was conducted with the approval of Osaka Prefecture University Animal Care and Use Committee (21–30). The sample was loaded on 1-mm thick sodium dodecyl sulfate polyacrylamide gels and was separated by electrophoresis. Proteins were then transferred on to 0.45 μm nitrocellulose membrane (Bio-Rad, Hercules, CA) and was probed with goat polyclonal anti-canine transferrin antibody (ThermoFisher Scientific, Waltham, MA). The membrane was washed and incubated with HRP-conjugated donkey anti-goat antibody (Peroxidase AffiniPure Donkey Anti-Goat IgG: Jackson ImmunoResearch Inc., West Grove, PA) Western blotting was performed to detect canine transferrin of serum and CSF of the normal dog, and also the leaked nasal fluid from this case. The characteristic pattern of transferrin is shown in Figure 3. Serum used as negative control showed only one band of transferrin, whereas two bands of transferrin were detectable in the CSF sample. In the leaked nasal fluid in this case, both bands were present.

After antimicrobial treatment (cephalexin; Riken Vets Pharma, Saitama, Japan, 25 mg/kg, BID, for 3 weeks), the cytologic evaluation showed non-infectious inflammation. The owner declined surgical treatment to stop CSF leakage. Over the following 5 months, the dog remained normal in its general condition, and epileptic seizures did not worsen. On the 167th day a sudden worsening of its general condition was observed without seizures, and the dog died. No pathological autopsy was performed after death, and the precise cause of death was not identified.

## Discussion

There have been only a few reports of diagnosis and treatment of CSF rhinorrhea in dogs and cats (7, 9). In the present case, CSF rhinorrhea was suspected based on clinical signs and MRI findings. Transferrin is an iron transport protein. Two isoforms of transferrin are known to exist. These are serum-type transferrin (β1) and brain-type transferrin (β2). The β1 isoform is present in serum and CSF, and the β2 isoform is found specifically in CSF (6, 10). Serum used as negative control showed only the β1 isoform, whereas β1 and β2 were both detectable in the CSF sample. In the nasal fluid leaked in this case, both isoforms of transferrin were suggestive for CSF rhinorrhea as the origin of the nasal discharge. These observations of transferrin isoforms in the nasal discharge suggested that CSF rhinorrhea was present.

In humans, commonly used modalities for confirming CSF rhinorrhea include CT, MRI, endoscopy, CT and MRI cisternography (4, 11, 12). High resolution CT reportedly has an accuracy of 92%, a sensitivity of 92% and a specificity of 100% in detecting CSF rhinorrhea in humans (3). Bony high resolution CT is valuable in identifying the site of a CSF fistula as a dural and osseous defect. This is believed to be effective in diagnosing acquired CSF rhinorrhea, due for instance to trauma. Induced partial defect of the left cribriform plate was observed in the present case, and this is regarded as useful in detecting CSF rhinorrhea. Mostafa et al. studied the role of MRI in the diagnosis of CSF leaks in humans. In their study of 20 human patients with suspected CSF rhinorrhea, T2-weighted MRI shows CSF as a bright signal, although spatial resolution is poor, as is the depiction of bony details. Fat-suppressed T2-weighted MRI detected a CSF-like density in 18 cases, with a sensitivity of 88.9%. Superimposition of the CTs and MRIs accurately localized the site of CSF leakage in 17 of 19 cases with a sensitivity of 89.7% (13). MR cisternography is a robust technique for demonstrating CSF leaks. It was first employed in 1986 by Dichiro (14), using cisternal injection of contrast material to enhance CSF in dogs. With advances in MR technology, however, the intrathecal injection is discouraged. Almost all studies of MR cisternography are performed using 1.5 tesla and high strength MRI scanners. MRI cisternography has an accuracy of 92%, a sensitivity of 89% and a specificity of 100% in humans (3, 15). Leakage of CSF was not clearly detected on MRI in the present case, because it is difficult to identify the irregular lesion in the nasal cavity around the left cribriform plate. The left cribriform plate was more irregular than the right cribriform plate, which may indicate a defect in the cribriform plate. The irregular lesion in the nasal cavity around the left cribriform plate may be due to CSF leaking into the left nasal cavity.

Detection of glucose in nasal fluid is a classical method for testing CSF leakage in humans. The normal concentration of glucose in nasal fluid is less than 10 mg/dL, and, a concentration greater than 30 mg/dL glucose suggests severe rhinitis or CSF leakage into the nasal cavity in humans (16, 17). The glucose concentration of CSF in dogs is normally 80% of the blood level (18). One study found that the glucose concentration of CSF varied from 53 to 104 mg/dL in healthy dogs (19). Unfortunately, this test has high false positive and negative rates in humans, depending on the patient's other medical conditions (20). With a sensitivity of 94–100% and a specificity of 98–100%, detection of brain-type transferrin assay has become the gold standard technique in diagnosis of CSF leakage in humans (21–23), verified by the detection of a further band distinct from serum-type transferrin (24, 25). No diagnostic criteria have been established for CSF leakage into the nasal cavity in veterinary medicine. In the present study, diagnosis of CSF rhinorrhea could usefully focus on the lesion cribriform plate, and glucose and β2 transferrin in nasal discharge.

The most common clinical signs in humans are a leak of clear and watery fluid from the nose and ear, with positional dependency (17). Most human patients with cerebral CSF leak complain of headache, neck pain, nausea, and vomiting. These conditions are believed to be related to CSF hypotension (26). Recurrent bacterial meningitis due to CSF rhinorrhea make the clinical signs worse (16, 27). Meningitis is found in patients with persistent CSF rhinorrhea, reportedly ranging from 10 to 37% (4). The most common pathogens of meningitis are Streptococcus and Hemophilus influenza. In the present case, epileptic seizure and continuous nasal discharge were observed. Meningitis in the forebrain region may be responsible for these clinical signs in view of the imaging results. The nasal discharge may have gone unnoticed or been considered unimportant for a fairly long time, and infection at this later age may have precipitated the problems. If dogs present with chronic nasal discharge, CSF leakage into the nasal discharge should be considered as a differential diagnosis.

There are two main ways of treating CSF leakage into the nasal cavity. These are conservative management and surgical repair. The treatment of CSF rhinorrhea in humans depends on its cause, and surgery is not indicated only in the acute phase after traumatic brain injury (16). The success rate of closure by endoscopic nasal reconstruction has reportedly improved in humans (12, 17). Other techniques include multiple closure using fat, fascia, cartilage, artificial dura mater, and nasal mucosa, and a closure technique using a stalked mucosal valve that takes nutritional blood vessels into account (16). In small animal medicine, some meningoencephalocele cases showed CSF rhinorrhea, and there are sporadically reports describing surgical repair of meningoencephaloceles (28–30). Further studies are needed to clarify what surgical option is best and under what conditions it should be performed. In addition, as there are currently no diagnostic criteria for CSF rhinorrhea, a pathological autopsy is necessary to confirm its diagnosis.

## Conclusion and Clinical Relevance

CSF rhinorrhea should be included in the differential diagnosis when persistent nasal discharge and forebrain signs are observed. Detection of nasal glucose and brain-type transferrin would be useful for diagnosing CSF rhinorrhea in dogs.

## Data Availability Statement

The original contributions presented in the study are included in the article, further inquiries can be directed to the corresponding author.

## Ethics Statement

The animal study was reviewed and approved by Osaka Prefecture University Animal Care and Use Committee. Written informed consent was obtained from the owners for the participation of their animals in this study.

## Author Contributions

KKi, HN, RK, YN, KKu, TT, and SS assisted with the diagnosis of this case and participated in clinical case management. HN and HA participated in the review and editing of the manuscript. All authors contributed to the article and approved the submitted version.

## Funding

Funding was provided in part by Grant-in-Aid for Scientific Research C (Grant No. 19K06405) from the Japan Society for the Promotion of Sciences.

## Conflict of Interest

The authors declare that the research was conducted in the absence of any commercial or financial relationships that could be construed as a potential conflict of interest.

## Publisher's Note

All claims expressed in this article are solely those of the authors and do not necessarily represent those of their affiliated organizations, or those of the publisher, the editors and the reviewers. Any product that may be evaluated in this article, or claim that may be made by its manufacturer, is not guaranteed or endorsed by the publisher.

## References

[1] Adoga AA. Cerebrospinal fluid rhinorrhea–an overview. Niger J Med. (2009) 18:244–9. 10.4314/njm.v18i3.51163 20120638

[2] Bathla G Moritani T. Imaging of cerebrospinal fluid leak. Semin Ultrasound CT MR. (2016) 37:143–9. 10.1053/j.sult.2015.12.002 27063664

[3] Vemuri NV Karanam LSP Manchikanti V Dandamudi S Puvvada SK Vemuri VK. Imaging review of cerebrospinal fluid leaks. Indian J Radiol Imaging. (2017) 27:441–6. 10.4103/ijri.IJRI_380_16 29379240 PMC5761172

[4] Oakley GM Alt JA Schlosser RJ Harvey RJ Orlandi RR. Diagnosis of cerebrospinal fluid rhinorrhea: an evidence-based review with recommendations. Int Forum Allergy Rhinol. (2016) 6:8–16. 10.1002/alr.21637 26370330

[5] Sharma SD Kumar G Bal J Eweiss A. Endoscopic repair of cerebrospinal fluid rhinorrhoea. Eur Ann Otorhinolaryngol Head Neck Dis. (2016) 133:187–90. 10.1016/j.anorl.2015.05.010 26776882

[6] Shelesko EV Kravchuk AD Kapitanov DN Chernikova NA Zinkevich DN. A modern approach to the diagnosis of nasal liquorrhea. Z Vopr Neirokhir Im N N Burdenko. (2018) 82:103–11. 10.17116/neiro2018823103 29927432

[7] Lazzerini K Gutierrez-Quintana R José-López R McConnell F Gonçalves R McMurrough J. Clinical features, imaging characteristics, and long-term outcome of dogs with cranial meningocele or meningoencephalocele. J Vet Intern Med. (2017) 31:505–12. 10.1111/jvim.14638 28247440 PMC5354015

[8] Rosenblatt AJ Scrivani PV Caserto BG Ruby RE Loftus JP de Lahunta A. Imaging diagnosis–meningoencephalitis secondary to suppurative rhinitis and meningoencephalocele infection in a dog. Vet Radiol Ultrasound. (2014) 55:614–9. 10.1111/vru.12105 24103047

[9] Nozue Y Yamazaki M Nakata K Nakano Y Yuki G Kimata A. Surgical treatment for intranasal meningoencephalocele in a cat. Front Vet Sci. (2020) 7:532. 10.3389/fvets.2020.00532 32974400 PMC7472124

[10] Oh JW Kim SH Whang K. Traumatic cerebrospinal fluid leak: diagnosis and management. Korean J Neurotrauma. (2017) 13:63–7. 10.13004/kjnt.2017.13.2.63 29201836 PMC5702760

[11] Eljazzar R Loewenstern J Dai JB Shrivastava RK Iloreta AM Jr. Detection of cerebrospinal fluid leaks: is there a radiologic standard of care? A systematic review. World Neurosurg. (2019) 127:307–15. 10.1016/j.wneu.2019.01.299 30797912

[12] Vladimir K Petar V Ljiljana V Slobodan S Danijela D Vladimir P. Endoscopic repair of cerebrospinal fluid rhinorrhea. Braz J Otorhinolaryngol. (2017) 83:388–93. 10.1016/j.bjorl.2016.04.024 27320657 PMC9442702

[13] Mostafa BE Khafagi A. Combined HRCT and MRI in the detection of CSF rhinorrhea. Skull Base. (2004) 14:157–62. 10.1055/s-2004-832259 16145599 PMC1151686

[14] Di Chiro G Girton ME Frank JA Dietz MJ Gansow OA Wright DC. Cerebrospinal fluid rhinorrhea: depiction with MR cisternography in dogs. Radiology. (1986) 160:221–2. 10.1148/radiology.160.1.3715036 3715036

[15] Tuntiyatorn L Laothammatas J. Evaluation of MR cisternography in diagnosis of cerebrospinal fluid fistula. J Med Assoc Thai. (2004) 87:1471–6. 15822543

[16] Matsuwaki Y Omura K Mori R Joki T Ishida M Moriyama H. Diagnosis and treatment of nasal cerebrospinal rhinorrhea (in japanese). Otorhinolaryngology. (2010) 53:300–10. 10.11453/orltokyo.53.300

[17] Choi D Spann R. Traumatic cerebrospinal fluid leakage: risk factors and the use of prophylactic antibiotics. Br J Neurosurg. (1996) 10:571–5. 10.1080/02688699646880 9115653

[18] Chrisman CL. Cerebrospinal fluid analysis. Vet Clin North Am Small Anim Pract. (1992) 22:781–810. 10.1016/S0195-5616(92)50077-8 1641918

[19] Kim IH Jung DI Yoo JH Kang BT Park C Park HM. Cerebrospinal fluid analysis in 13 clinically healthy Beagle dogs: hematological, biochemical and electrophoretic findings. Korean J Vet Res. (2008) 48:105–10.

[20] Chan DT Poon WS Ip CP Chiu PW goh KY. How useful is glucose detection in diagnosing cerebrospinal fluid leak? The rational use of CT and Beta-2 transferrin assay in detection of cerebrospinal fluid fistula. Asian J Surg. (2004) 27:39–42. 10.1016/S1015-9584(09)60242-6 14719513

[21] Warnecke A Averbeck T Wurster U Harmening M Lenarz T Stöver T. Diagnostic relevance of β2-transferrin for the detection of cerebrospinal fluid fistulas. Arch Otolaryngol Head Neck Surg. (2004) 130:1178–84. 10.1001/archotol.130.10.1178 15492165

[22] Haft GF Mendoza SA Weinstein SL Nyunoya T Smoker W. Use of beta-2-transferrin to diagnose CSF leakage following spinal surgery: a case report. Iowa Orthop J. (2004) 24:115–8. 15296217 PMC1888406

[23] Fransen P Sindic CJM Thauvoy C Laterre C Stroobandt G. Highly sensitive detection of beta-2 transferrin in rhinorrhea and otorrhea as a marker for cerebrospinal fluid (C.S.F.) leakage. Acta Neurochir. (1991) 109:98–101. 10.1007/BF01403002 1858539

[24] Papadea C Schlosser RJ. Rapid method for beta2-transferrin in cerebrospinal fluid leakage using an automated immunofixation electrophoresis system. Clin Chem. (2005) 51:464–70. 10.1373/clinchem.2004.042697 15608153

[25] Görögh T Rudolph P Meyer JE Werner JA Lippert BM Maune S. Separation of beta2-transferrin by denaturing gel electrophoresis to detect cerebrospinal fluid in ear and nasal fluids. Clin Chem. (2005) 51:1704–10. 10.1373/clinchem.2005.054916 16020492

[26] Kunihiro T Soma K. Dizziness and other symptoms of cerebrospinal fluid leakage and insights into their pathogenesis. Equil Res. (2014) 73:174–86. 10.3757/jser.73.174

[27] Takao N Sakurai K Hino S Yamano Y. A case of nasal discharge resulting in recurrent bacterial meningitis. Rinsho Shinkeigaku. (2021) 61:177–81. 10.5692/clinicalneurol.cn-001505 33627580

[28] Martle VA Caemaert J Tshamala M Van Soens I Bhatti SF Gielen I. Surgical treatment of a canine intranasal meningoencephalocele. Vet Surg. (2009) 38:515–9. 10.1111/j.1532-950X.2009.00534.x 19538674

[29] Dewey CW Brewer DM Cautela MA Talarico LR Silver GM. Surgical treatment of a meningoencephalocele in a cat. Vet Surg. (2011) 40:473–6. 10.1111/j.1532-950X.2011.00813.x 21418253

[30] Mojarradi A Van Meervenne S Suarez-Bonnet A De Decker S. Diagnosis, treatment and postsurgical complications in a dog with epileptic seizures and a naso-ethmoidal meningoencephalocele. Acta Vet Scand. (2021) 63:26. 10.1186/s13028-021-00591-1 34238330 PMC8268512

